# Utility of the Xpert MTB/RIF Assay for Diagnosis of Tuberculous Meningitis

**DOI:** 10.1371/journal.pmed.1001537

**Published:** 2013-10-22

**Authors:** David R. Boulware

**Affiliations:** Division of Infectious Diseases and International Medicine, Department of Medicine, University of Minnesota, Minneapolis, Minnesota, United States of America

## Abstract

David Boulware discusses the challenges of diagnosing tuberculous meningitis and the implications of the study by Patel and colleagues using the Xpert MTB/RIF assay for diagnosis.

*Please see later in the article for the Editors' Summary*

**Linked Research Article**

This Perspective discusses the following new study published in *PLOS Medicine*:

Patel VB, Theron G, Lenders L, Matinyenya B, Connolly C, et al. (2013) Diagnostic Accuracy of Quantitative PCR (Xpert MTB/RIF) for Tuberculous Meningitis in a High Burden Setting: A Prospective Study. PLoS Med 10(10): e1001536. doi:10.1371/journal.pmed.1001536

Vinod Patel and colleagues evaluate the sensitivity and specificity of quantitative PCR using Xpert MTB/RIF for diagnosis of TB meningitis in the high-burden setting of South Africa.

Tuberculous meningitis (TBM) is characterized by copious cerebrospinal fluid (CSF) inflammation and yet few *Mycobacterium tuberculosis*. This combination creates a disease that is notoriously difficult to definitively diagnose. In this week's issue of *PLOS Medicine*, Patel and colleagues report the diagnostic performance of the GeneXpert system's Xpert MTB/RIF assay for the diagnosis of TBM in a cohort of 204 South African, predominantly HIV-infected, adults presenting with suspected meningitis of whom 59 had definitive TBM [1]. The Xpert MTB/RIF assay's overall sensitivity was 62%, and specificity was 95%. The performance was better using larger volumes of centrifuged CSF among HIV-infected persons with sensitivity of approximately 80% and excellent specificity for microbiologically confirmed TBM. Xpert MTB/RIF performance was less impressive using uncentrifuged CSF with a sensitivity of ≤50%, and Xpert MTB/RIF performance was negligible in HIV-uninfected persons.

## What Is GeneXpert?

The GeneXpert System (Cepheid) is a single use cartridge-based real-time PCR fully automated system that performs sample decontamination, sonication, automated nucleic acid amplification, and fluorescence-based quantitative PCR [2]–[4]. The Xpert MTB/RIF assay, developed by David Alland, detects *M. tuberculosis* DNA in approximately 2 hours with minimal hands-on time [3]. This new technology was endorsed by the World Health Organization in December 2010, and as of June 30, 2013, a total of 1,402 GeneXpert instruments and over 3 million Xpert MTB/RIF cartridges have been procured in the public sector in 88 countries [5]. The concessional pricing is US$9.98 per cartridge for 145 low- and middle-income countries [6]. The same GeneXpert platform also can be used for a variety of US Food and Drug Administration (FDA)-approved testing (e.g., influenza, *Clostridium difficile*, methicillin-resistant *Staphylococcus aureus*).

## What Is the Performance of Xpert MTB/RIF?

There is a rapidly emerging literature regarding the performance of the Xpert MTB/RIF assay. Fundamentally, the sensitivity depends on the burden of organisms and thereby the target DNA present in the specimen. The published Xpert MTB/RIF detection threshold is approximately 100–130 colony forming units (cfu)/ml of sample [2],[3]. Patel and colleagues observed a similar threshold of 80–100 cfu/ml of CSF in this study [1]. In comparison, the detection threshold is <10 cfu/ml for mycobacterial liquid culture and is >5,000 cfu/ml for Ziehl-Neelsen staining for acid fast bacilli (AFB) via standard microscopy in sputum [7]–[9]. In real world clinical terms, this means 98%–99% detection by Xpert MTB/RIF of AFB smear-positive pulmonary TB, and approximately 75% detection of smear-negative, culture-positive pulmonary TB [3],[10],[11].

The threshold of detection is a key principle. The Xpert MTB/RIF test performs better when there is a larger burden of infectious organisms present in the specimen being tested. Yet, TB meningitis is a paucibacillary condition with few organisms. More organisms are likely present when the host is immunocompromised, or when a larger input volume is used for the test. Thus specimen centrifugation can compensate and should improve diagnostic yield, as demonstrated by a 35% improvement in sensitivity in this study [1].

The prior data on Xpert MTB/RIF testing of CSF are limited. In India, the Xpert MTB/RIF assay detected two of seven culture-positive specimens using an input volume of ∼1 ml [12]. In an Italian study, 11 of 13 TBM patients were detected by Xpert MTB/RIF using an input volume of 2 ml into the cartridge without the standard N-acetyl-L-cysteine-sodium hydroxide (1% NALC-NaOH) decontamination and mucolytic step (i.e., GeneXpert Sample Reagent) [13]. This sample reagent was designed for sputum samples. Numerous commercially available PCR assays exist for other pathogens (e.g., herpes simplex PCR) without such a decontamination step [13], and the necessity of using the sample reagent for non-bloody CSF is unclear.

## Public Health Significance

Although two commercial TB PCR tests previously existed [14], the innovation is that the GeneXpert platform is fully automated and is being rolled out in low- and middle-income countries. Thus, GeneXpert is an actual technology that can be—and is being—widely used globally. However, immediate implementation of a US$10 Xpert MTB/RIF assay for all cases of meningitis is unwise and unsustainable. Further research is needed on how best to incorporate the Xpert MTB/RIF test into diagnostic testing for meningitis, to ensure that it is a cost-effective intervention that improves health and does not waste resources.

Patel and colleagues modeled a clinical score to predict who had such high pretest probability of TBM that Xpert MTB/RIF was unnecessary to perform [1]. Yet health systems also need the opposite, a clinical score or algorithm to identify who has such low pretest probability that they do not require testing. Several investigators have developed meningitis diagnostic algorithms, yet broader validation is needed [15]–[17]. Ordering comprehensive testing of all the available diagnostic tests for every patient with suspected meningitis, including Xpert MTB/RIF testing, is 3–4-fold more expensive, without any better diagnostic yield than a targeted stepwise approach [15].

## Key Principles of TB Meningitis Diagnosis

Despite molecular diagnostics, there remain a number of key pieces of information that inform clinicians as to the likelihood of a TBM diagnosis, so as to target testing in a cost-effective manner. The first is history. TBM is a subacute illness. Symptoms <6 days are atypical for TBM, yet near universal for bacterial meningitis [16]. Second is the immunology of the patient. Immunosuppression due to HIV/AIDS or age (e.g., infants, elderly) are key drivers of TBM, and immunosuppression increases the bacillary burden of *M. tuberculosis* organisms, likely increasing the diagnostic yield of molecular testing. In the current study, the GeneXpert performed poorly among HIV-uninfected persons, and the performance in children is unknown. Third is the CSF profile. TBM is classically a lymphocytic meningitis (i.e., >30%–50% lymphocytes in >90% of persons [16],[18]); with a low CSF glucose of <60% of serum glucose or an absolute CSF glucose concentration <2·2 mmol/l (<40 mg/dl) in >92%–95% [18]–[20].

In HIV-infected adults, the clinical history and CSF profile overlap extensively with meningitis due to *Cryptococcus neoformans*, and cryptococcal meningitis is overall the most common meningitis etiology in adults in sub-Saharan Africa [15]. Thus before a US$10 Xpert MTB/RIF test is performed for a less common condition, a US$2 cryptococcal antigen lateral flow assay should likely be performed for a more frequent condition [15].

If there is insufficient CSF volume available for testing (i.e., <3 ml), in a clinically stable patient treated presumptively for bacterial meningitis a repeated lumbar puncture in 48 hours is likely a better strategy than sub-optimal Xpert MTB/RIF testing using a limited volume. A repeat lumbar puncture can collect a sufficiently large volume as well as reassess CSF glucose. At 48 hours, the CSF glucose should have risen by >100% of the initial level in treated bacterial meningitis [16]. Persistently low CSF glucose levels at 48 hours coupled with excluding cryptococcal meningitis should prompt Xpert MTB/RIF testing and/or empiric anti-TB therapy [16].

Xpert MTB/RIF appears to be a highly useful test to “rule in” the diagnosis of TBM, yet the clinical acumen of physicians remains a necessity for the wise use of any new diagnostic test. Careful application of these new diagnostic tools should improve clinicians' ability to deliver timely, cost-effective care to patients with suspected TBM throughout the world, an approach that future studies should systematically evaluate.
